# Rupture of an aneurysm of the gastroduodenal artery mimicking a tumor of the head of the pancreas in a case report.

**DOI:** 10.1016/j.radcr.2021.12.061

**Published:** 2022-01-15

**Authors:** Geraud Akpo, Ahma Dia, Nfally Badji, Hamidou Deme, Boucar Ndong, Marcel Mbarga, Ibrahima Niang, Abdoulaye Dione Diop, Sokhna Ba, El hadji Niang

**Affiliations:** a Radiology and medical imaging department of Aristide Le Dantec Hospital (Dakar-Senegal); b Radiology and medical imaging department of Dalal Jam hospital (Dakar-Senegal); c Radiology and medical imaging department of Fann Hospital (Dakar-Senegal); d Nuclear medicine department of Dalal Jam hospital (Dakar-Senegal)

**Keywords:** Aneurysm, Gastruodenal, Rupture

## Abstract

Gastroduodenal artery aneurysms have a low incidence of less than 1.5% of all splanchnic aneurysms. Rupture is frequent and life-threatening. The diagnosis is made by CT scan and by coeliac angiography, which also plays a therapeutic role in stable or stabilized patients. Surgery remains the treatment of choice in case of hemodynamic instability. We reviewed the case of a ruptured aneurysm of the gastroduodenal artery mimicking a tumor of the pancreas’ head in a patient who died following a hemorrhagic shock.

## Introduction

Gastroduodenal artery aneurysms are rare, corresponding to less than 1.5% of splanchnic artery aneurysms [1].Their rupture is frequent, and is mostly presented as internal or digestive hemorrhage, and is associated with a mortality rate that can reach 40% [2]. A CT scan with injection and coeliac arteriography allows the diagnosis of this condition, which can be treated surgically or radiologically [2]. We reviewed the case of a patient with a ruptured gastroduodenal aneurysm mimicking a tumor of the pancreatic head.

## Observation

Patient data: 51-year-old patient, active smoker (8 packs per year), ethylic, non-HTA, non-diabetic, received on account of cholestasis jaundice.

Clinical findings: oedemato-ascitic syndrome, generalized weakness and hemodynamically stable.

Diagnostic approach: Hematology and biochemistry findings show microcytic hypochromic anemia with a hemoglobin level of 8.4 g/dl, high neutrophils, leukocytosis with a positive C-RP of 24 mg/dl, a biological cholestasis syndrome with total and conjugated hyper-bilirubinemia, increased PAL and GGT and hepatic transaminases.

**Fig. 1:**
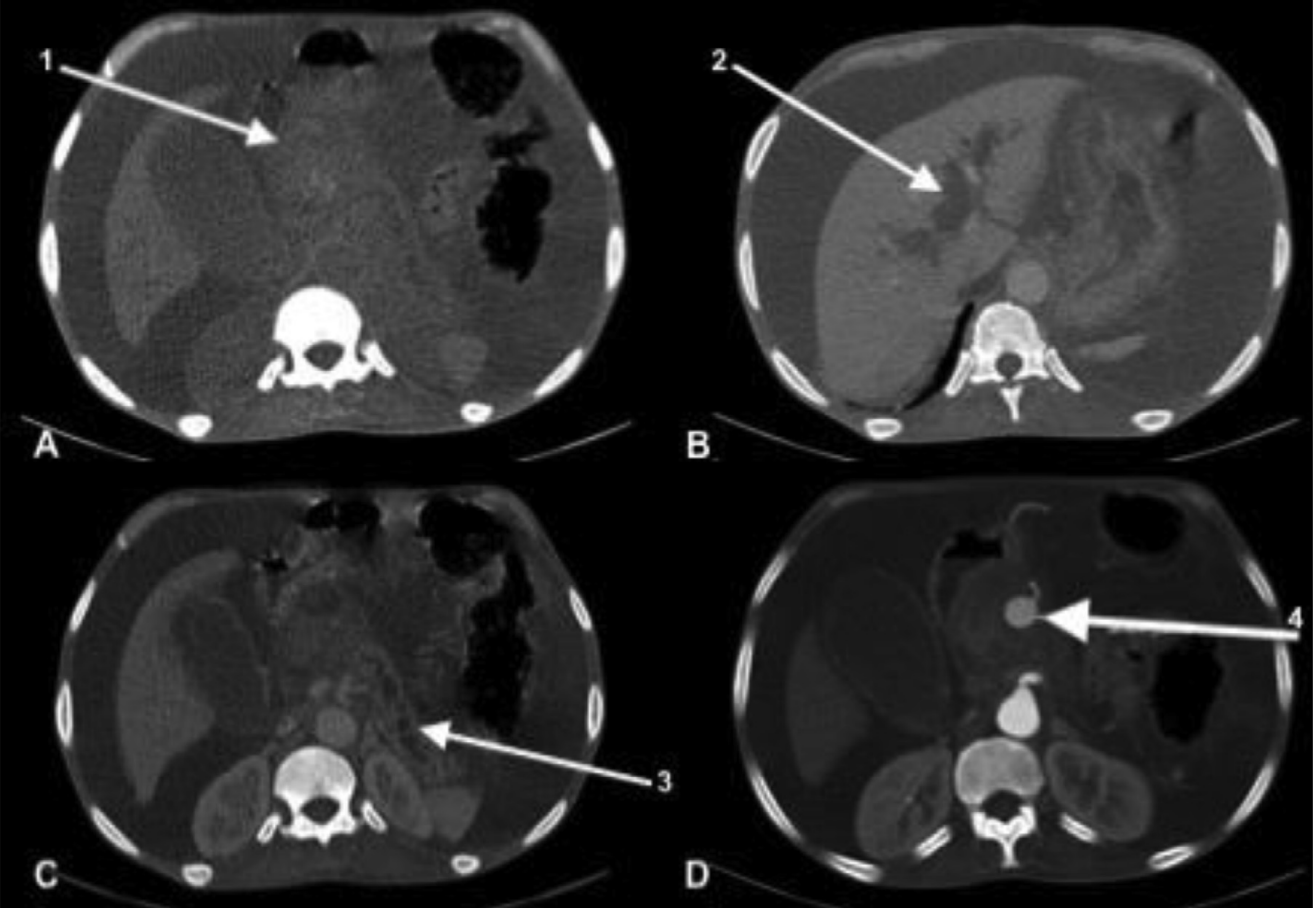
Abdominal CT scan showing in axial sections without injection (A) and with injection at the arterial and portal times (B, C and D) a pseudo-mass of the head of the pancreas (1) with dilatation of the intrahepatic bile ducts (2) and of the Wirsung duct (3). This pseudo-mass corresponds to a hematoma due to rupture of an aneurysm of the gastroduodenal artery (4)

**Fig. 2:**
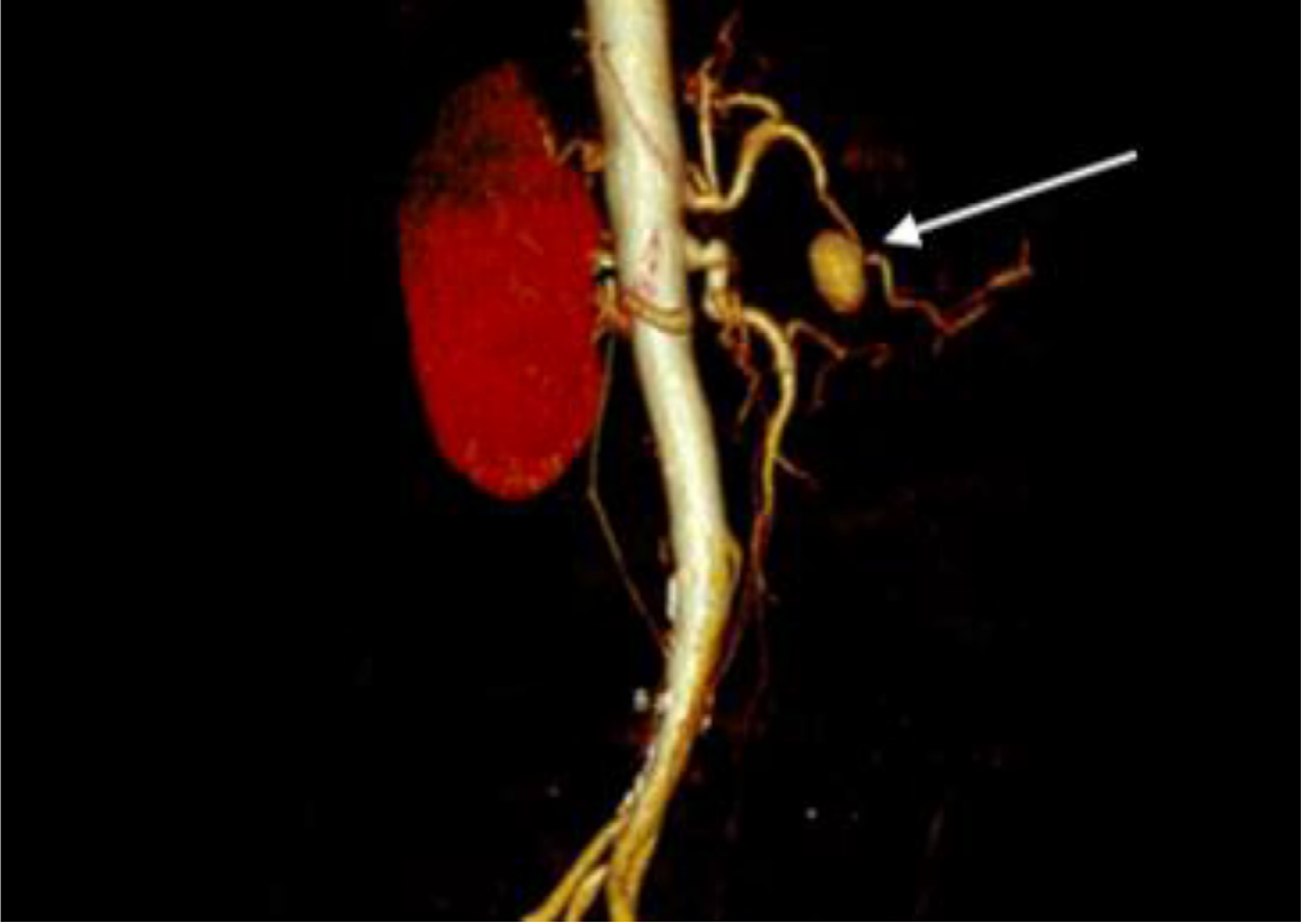
VRT reconstruction of an abdominal angioscan showing an aneurysmal sac of the gastroduodenal artery with a narrow neck

Abdominal ultrasound showed dilatation of the intra- and extra-hepatic bile ducts without visualized obstruction. An abdominal CT scan had shown the presence of a spontaneously dense, heterogeneous, poorly bounded formation appearing to grow partially over the head of the pancreas (Fig. 1A). It is associated with dilatation of the Wirsung duct, intra and hepatic bile ducts, a large gallbladder and a large peritoneal fluid effusion (Fig. 1 B and C). Contrast injection revealed at arterial phase, the presence of an 18x15mm saccular aneurysm of the gastroduodenal artery with a 2.2mm neck within the unenhanced mass (Figs. 1D and 2). The patient died 48 hours after the examination due to hemorrhagic shock.

## Discussion

Gastroduodenal artery aneurysms are mostly asymptomatic, discovered incidentally on imaging, or present with a vague symptomatology of atypical abdominal pain or a pulsatile mass on clinical examination [3]. Rupture is the main inaugural mode in more than 50% of cases [4] and may be responsible for internal hemorrhage (hemo-peritoneum, hemo-retroperitoneum), or externally through the digestive tract (hematemesis, melena), followed or not by hemodynamic shock. A compressive effect of the peripancreatic hematoma can give non-specific symptoms such as cholestasis jaundice [5,6] which was one of the signs present in the patient. This form of obstructive pseudotumor with dilatation of the intra- and extrahepatic bile ducts and the main pancreatic duct is rarely described in the literature.

According to Green et al [7], the occurrence of these aneurysms is consecutive to pancreatitis in which, the autodigestion of the gland by enzymatic activation extends to the walls of the peripancreatic vessels and gives false aneurysms. The interrogation did not report in the patient's history a previous pancreatitis picture unless it went unnoticed. The presence of pancreatic calcifications on CT scan (better visualized in spontaneous contrast) or even on APS (unprepared abdominal radiograph) could have suggested chronic pancreatitis. These signs were absent in this case.

True aneurysms are very rare and the etiological factors that have been suggested are atherosclerosis, periarteritis nodosa, and fibromuscular dysplasia [8].

Other factors may be associated such as trauma, stenosis of the celiac trunk, alcohol abuse, previous cholecystectomy, Marfan's syndrome or hepatic cirrhosis [2]. A notion of ethylism was reported in the anamnesis.

A few atheromatous calcified plaques were also described during the CT scan. Other etiological factors were not reported.

The work of Moore et al [5] showed that the diagnosis is based on abdominal angioscan which allows characterization of the aneurysm (site, size, saccular, fusiform morphology, neck), the search for other aneurysmal locations, and complications, in particular rupture or compressive effect, and possible associated anomalies.

Compression of the bile ducts was due to a perianeurysmal hematoma leading to dilatation of the latter. The main pancreatic duct of Wirsung was also dilated. It was therefore a mass located at the level of the pancreatic cephalic pole responsible for a bichannel dilatation. It is thanks to the injection at the arterial phase that it allowed us to unmask an aneurysm by specifying its shape, size and location (gastro-duodenal artery). The defect of the mass enhancement around the aneurysm confirms its nature (hematoma). Coeliomy arteriography proposed in stable or stabilized patients goes further beyond its diagnostic role, it allows treatment by embolization [5]. Endovascular methods are either direct, by embolization of the aneurysm with coils, or indirect by embolizing all the aneurysmal arterial afferences and efferences, as well maintaining arterial patency by placing a covered stent if the arterial anatomy allows it [9]. The success rate is higher than 90% and the main complication is recanalization of the aneurysm, showing the importance of post-treatment follow-up, which should, preferably, be performed by magnetic resonance imaging [9].

The patient could not benefit from an emergency embolization because it was not available in the “Thies region” located about 70 km from Dakar.

Surgical management consists of ligation of the gastroduodenal artery after complete resection of the aneurysm [5]. It is the treatment of choice in case of rupture or hemodynamic instability as observed in our patient or in the presence of an anatomical variant hostile to endovascular treatment [3]. However, the morbi-mortality linked to surgery is very high and, more and more, certain teams such as that of Kueper [10] propose an interventional radiological treatment in case of rupture if the hemodynamic conditions of the patient allow it. Embolization can also allow rapid and easy hemostasis and leave time to prepare for surgery under better conditions. These techniques offer many advantages compared to surgical hemostasis, they avoid the risks of extensive surgical dissection and limit the resection of surrounding organs for hemostatic purposes. As a result, they significantly reduce the length of stay in the intensive care unit and the overall hospital stay [11].

## Conclusion

This observation shows a rare mode of expression of a ruptured gastroduodenal artery aneurysm as a pseudotumor of the pancreatic head. It raises the interest of an early diagnosis for a rapid management by endovascular interventional techniques. Although there is an increasing trend towards their use, surgery remains the treatment of choice in cases of hemodynamic instability.
